# Phylogenetic analysis of Columbid herpesvirus-1 in rock pigeons, birds of prey and non-raptorial birds in Poland

**DOI:** 10.1186/1746-6148-9-52

**Published:** 2013-03-21

**Authors:** Grzegorz J Woźniakowski, Elżbieta Samorek-Salamonowicz, Piotr Szymański, Piotr Wencel, Marek Houszka

**Affiliations:** 1 National Veterinary Research Institute (NVRI), Department of Poultry Viral Diseases, Partyzantow 57 Avenue, Pulawy, 24-100, Poland; 2 Wild Animal Treatment and Rehabilitation Centre, Wroclaw University of Environmental and Life Sciences 55–106 Zawonia, Zlotowek 1, Poland; 3 Avi Expert Private Veterinary Practice, Gajowa 1, Lublin, 20-827, Poland; 4 Wroclaw University of Environmental and Life Sciences, Department of Pathology, CK, Norwida 31, Wroclaw, 50-375, Poland

**Keywords:** Columbid herpesvirus-1, Phylogenetic analysis, Real-time PCR, Sequence analysis, DNA-dependent polymerase

## Abstract

**Background:**

The identity of herpesviruses isolated in Europe from domestic pigeons (Columbid herpesvirus-1 - CoHV-1) as well as falcons and owls remains unknown. All these herpesviruses are antigenically and genetically related. The falcons and owls are thought to have become infected during the ingestion of pigeon meat thus suggesting the virus’s capacity to infect a wide range of hosts. The aim of the conducted study was to detect the occurrence of CoHV-1 and estimating the similarities and differences in the DNA-dependent DNA polymerase gene of herpesviruses isolated from domestic pigeons, birds of prey and non-raptorial free-ranging birds in Poland.

**Results:**

The study has shown the presence of CoHV-1 in 20.4% (18/88) in the examined birds. In case of one CoHV-1, infected Peregrine Falcon (*Falco peregrinus*), neurological signs were observed. Nucleotide sequencing of the DNA-dependent DNA polymerase gene, showed a high similarity among Polish strains (100%), independently from the species of the affected birds. Only one compared CoHV-1 strain - KP 21/23 originating from Germany showed a slightly lower similarity at a level of 99.1%. Further analysis has shown the identity of DNA-dependent DNA polymerase of CoHV-1 strains and other herpesviruses present in poultry as well as other birds ranged from 35.4 to 44.9%. Interestingly CoHV-1 infection was also confirmed for the first time in four non-raptorial birds.

**Conclusions:**

The current study has shown a high similarity of CoHV-1 strains and the possible transmission of herpesviruses between domestic rock pigeons and free-ranging birds including raptors and non-raptorial birds. Further studies focused on cloning and the analysis of the whole CoHV-1 genome which is needed to explain the role of the observed similarities and differences between field strains of columbid herpesviruses.

## Background

Herpesviruses of domestic rock pigeons and free-ranging birds cause a variety of clinical signs. Due to latent properties of *Herpesviridae* they may also remain a subclinical infection that influences the immune status of birds [1,2]. Herpesviruses of free-ranging birds fall into seven different species including herpesviruses of pigeons, falcons, vultures, owls and Psittacid herpesvirus (PsHV-1) affecting parrots [2-6]. CoHV-1 isolated from pigeons and falcons, presents similar antigenic features thus its differentiation by serological assays is not possible [7,8]. CoHV-1 has been primarily classified as member of *Betaherpesvirinae* due to its pathology and growth features. However, it has been further reclassified as an Alphaherpesvirus because of its close relation to the Marek’s disease virus in chickens (MDV) [9,10]. Meanwhile, MDV officially named as *Gallid herpesvirus 2* (MDV-1), shares common genetic properties with other herpesviruses isolated in poultry including the infectious laryngotracheitis virus - ILTV (*Gallid herpesvirus 1*), MDV serotype 2 – MDV-2 (*Gallid herpesvirus 3*), and the previously mentioned PsHV-1 infecting parrots. Infection with CoHV-1 spreads by direct contact with the infected birds as well as by consumption of infected pigeon meat by birds of prey [1,2]. Therefore, pigeons are thought to be the main source of infection also for free-ranging birds [11]. The observed clinical signs in pigeons are called Smadel’s disease [3,11] or fatal inclusion body hepatitis in falcons and owls [1,12-14]. Smadel’s disease is present in young squabs between ten and sixteen weeks old [1,3]. The birds may not present any clinical signs except depression, anorexia or conjunctivitis. In case of inclusion body hepatitis in falcons, the clinical signs are non-specific and may also be manifested by weakness and anorexia. The specific lesions observed during Smadel’s disease include upper respiratory tract inflammation and ulceration as well as hepatic and splenic necrosis [1,3]. In case of herpesvirus infection of falcons the lesions include small necrotic foci in liver, spleen, kidney, pharynx and bone marrow [14-16]. Neurological signs are also possible but rare [13]. The infection may also remain latent making pigeons susceptible to secondary infections with other viral pathogens [11,17]. The previously described infections with CoHV-1 were observed among *Accipitridae* family in Common Buzzards (*Buteo buteo*), Booted Eagles *(Hieraaetus pennatus)* and Cooper Hawks (*Accipiter Cooperi)*[13,14,16]. Owls (*Strigidae*), such as the great horned owl *(Bubo virginianus),* may also be affected by the same virus [4,5]. The study on CoHV-1 occurrence in free-ranging non-raptorial birds has not been previously described.

The study was done to explain the phylogenetic relationships of herpesviruses isolated from pigeons and free-ranging birds including birds of prey such as Peregrine Falcon (*Falco peregrinus*), Eurasian Kestrel (*Falco tinnunculus*), but also members of other families like Herring Gull (*Larus argentatus*) and Song Thrush (*Turdus philomelos*). The identity as well as transmission of herpesviruses isolated from free-ranging birds in Europe remains unclear. However since Alphaherpesviruses are characterised by their long survival in the environment, they pose a threat for all free-ranging birds, not only for raptors ingesting pigeon meat [18]. The study on CoHV-1 phylogenetic analysis among different free-ranging birds may provide additional information and evidence on the ubiquitous features of this virus.

## Methods

### Birds and samples

In total 88 dead birds were submitted by practitioners or bird rehabilitation centres during 2011–2012 for monitoring West Nile Virus (WNV) at the Department of Poultry Diseases at NVRI. During post-mortem, examination sections of brain were collected and examined for the WNV presence [19]. These brain sections were also used in this study for possible herpesvirus infection detection. The birds originated from different parts of Poland, predominantly form its east (Lublin) and south-west area (Wroclaw). The cause of death of the submitted birds was unknown. Due to the specific goal of the WNV surveillance project for its purposes only brains of free-ranging birds were submitted. Therefore, clinical history of birds from rehabilitation centres or practitioners was not available. However, in case of one female Peregrine Falcon (*Falco peregrinus*) submitted to our laboratory by dr Szymanski (Wild Animal Treatment and Rehabilitation Centre, Wroclaw) the clinical signs were observed. The bird was examined for the infection with AIV and aPMV-1 to exclude the influence of other associated infections.

### Virus isolation

The 10% (w/v) brain homogenates of field CoHV-1 isolates were used for virus propagation in secondary chicken embryo fibroblasts (CEF) prepared from 11-day old SPF chicken embryos (Lohmann Tierzucht, Cuxhaven, Germany). The cells were grown at 37°C and 5% CO_2_ in a MEM medium (Gibco, Paisley, United Kingdom) supplemented with 5% calf bovine serum (Gibco, Paisley, United Kingdom) and with a 1% (w/v) mixture of antibiotics (Antibiotic-antimycotic, Gibco, Paisley, United Kingdom). All strains were passaged three times then submitted to DNA extraction and real-time PCR. The cytophatic effect visible as clumping cells forming syncytia was observed starting from the 2^nd^ passage of the examined viruses. The cells were harvested 96 h p.i. when approximately 85% of the visible cytophatic effect (CPE) was observed. After triple freezing (−70°C), thawing (+20°C) the cell suspension was centrifuged at 600 × g, then the supernatant was aliquoted and stored at −70°C as stock solutions.

### Standard strains

Standard CoHV-1 strain GJW1126 originating from rock pigeon (*Columbia livia*) (Accession number: JX892997) and GJW1132 (Accession number: JX893002) from Peregrine Falcon (*Falco peregrinus*) were used (Department of Poultry Viral Diseases, National Veterinary Research Institute, Poland). These strains were used as a positive control for further real-time PCR development. The set of control strains represented by Marek’s disease virus (MDV) strain 31_07 (Department of Poultry Viral Diseases, NVRI, Poland), turkey herpesvirus (HVT) FC126 strain (Mérial, France), pigeon circovirus POL1 (PiCoV) (Department of Poultry Viral Diseases, National Veterinary Research Institute, Poland) and Fowl adenovirus 1 strain (FadV-1) (Charles River Laboratories International, Wilmington, United States of America) were also applied. The DNA templates for further analysis were extracted from 200 μL stock solutions of strains.

### DNA extraction

The total DNA for herpesvirus detection was extracted from 200 μL of virus stock solutions according to the procedure of the manufacturer for the QIAamp DNA Mini Kit (Qiagen, Hilden, Germany). The DNA was then stored and frozen at −80°C.

### Primer design

The primers were designed in Primer Express software version 2.0.1 (Applied Biosystems, Foster City, California, USA) on the basis of the conserved DNA-dependant DNA polymerase gene of CoHV-1 PO1W124 strain (Genbank: GQ478232) and were as follows CoHV1: 5′-GATGGCGGCCTGCTGTTTGT-3′, CoHV2 5′-CGCCGTGGACGACTTGCGT-3′. The primers were synthesised by Genomed Co. (Warsaw, Poland).

### Real-time PCR

The reaction was performed in 7500 Applied Biosystems system (Applied Biosystems, Foster City, California, USA). The reactions were set up on ice in 0.2 ml optical tubes with caps, using the Quantitect SYBR Green PCR Kit (Qiagen, Hilden, Germany). The reaction volume was 25 μL that contained: 12.5 μl of 2× QuantiTect SYBR Green PCR Master Mix, 40 pmol of each CoHV1 and CoHV2 primer, 1 μL of DNA template (~25 ng) and deionised water. All DNA templates were analysed in duplicates. The data were collected by Applied Biosystems software ver. 2.0.1 (Applied Biosystems, Foster City, California, USA). Reaction conditions were as follows: 50°C/2 min. (Uracil-DNA Glycosylase incubation), 95°C/15 min (initial denaturation), then 40 cycles of 95°C/1 min (exact denaturation) and 60°C/1 min (primer anealing and signal detection).

### Plasmid

The DNA template of standard GW1126 strain was used for amplification of DNA-dependent DNA polymerase. The pGW1126 plasmid was constructed by PCR product insertion (207 bp) into pGEM-T Easy vector (Promega, Fitchburg, Wisconsin, USA). The plasmid was cloned into DH5α cells (Invitrogen, San Diego, USA) with standard blue/white screening method. A single white colony with pGW1126 was used for the inoculation of 5 ml of liquid LB medium with ampicillin (100 μg/mL), and then the culture was incubated for 18 h at 37°C with shaking 225 rpm. The plasmid DNA was extracted from 5 ml of inoculated media using the Plasmid Mini Kit (Qiagen, Hilden, Germany) and used for the determination of real-time PCR sensitivity.

### Real-time PCR specificity and sensitivity

For the specificity test, DNA templates extracted from MDV strain 31_07, HVT FC126 strain, PiCoV POL1 strain and FadV-1 were applied. The sensitivity of the real-time PCR was examined using 5 ten-fold dilutions (100 ng – 10 pg) of the entire GW1126 strain (200 ng/μL) as well as 5 ten-fold dilutions of pGJW1126 plasmid (from 10^8^ to 10^1^ copies). The detection limit was determined as the highest dilution that resulted in a fluorescent signal plotted as a curve detected as cycle threshold (C_T_) value.

### DNA sequencing

The products obtained by real-time PCR were separated in a 1.5% agarose gel (Invitrogen, Green Island, New York, United States of America) then cut out and purified according to procedure of the QIAquick Gel Extraction Kit (Qiagen, Hilden, Germany). The amplicons were then sequenced on a GS FLX/Titanium sequencer (Roche, Branford, Connecticut, USA) by Genomed (Warsaw, Poland). Each product was sequenced in both forward and backward directions and then assembled into a single contig in Genious™ Software ver. 6.5.5 (Biomatters Ltd, Anzac Avenue, New Zealand). The sequences were aligned using Multiple alignment Genious™ algorithm with 93% cost matrix similarity and BOOTSTRAP analysis with 1000 repeats. To compare the obtained sequences 7 additional NCBI GeneBank entries of DNA-dependent DNA polymerase from CoHV-1 as well as other herpesviruses of poultry (MDV-1, MDV-2, HVT) and parrots (PsHV-1) were used. The results were also compared with the related human herpesvirus 1 (Accession number: JF810822) to show true phylogeny of the examined CoHV-1 Polish strains. The results were plotted as a similarity matrix and tree showing the phylogenetic distances and relationships between the analysed strains.

## Results

### Birds and clinical signs

**Figure 1:**
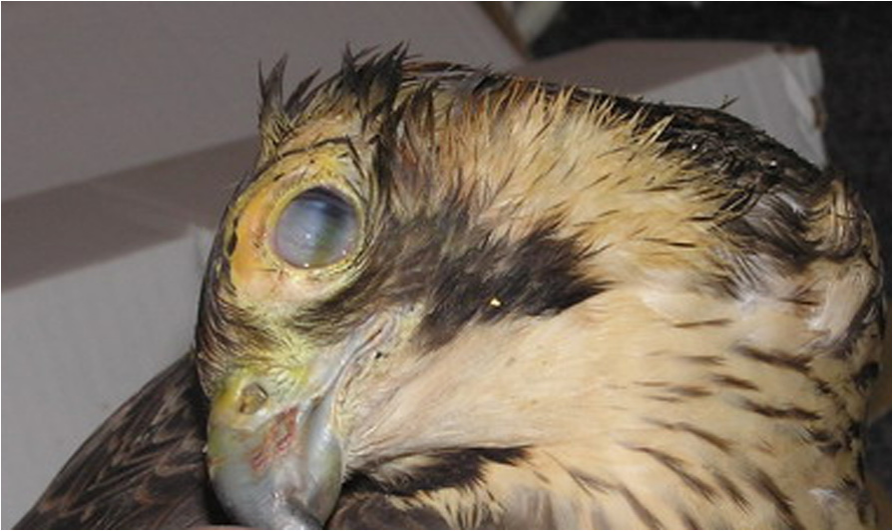
**Clinical signs observed in young female of Peregrine Falcon** (***Falco peregrinus***) **showing *torticollis *and keratitis.**

The cause of death for 88 birds used in this study was unknown since they were collected by practitioners for the monitoring study on WNV. In the case of one female Peregrine Falcon, clinical signs including keratitis and *torticollis* were observed (Figure 1). The Peregrine Falcon female from the rehabilitation centre was fed with pigeon meat, which further suggested the suspicion of herpesvirus infection, by ingestion. The bird died after the two weeks long treatment. The bird was also examined for possible infection with AIV, aPMV-1 and mechanical damage.

### Virus isolation

The conducted virus isolation was successful in the case of 18 strains. The viruses formed cytophatic effect starting from the 2^nd^ passage in CEF cell cultures.

### Real-time PCR

**Figure 2:**
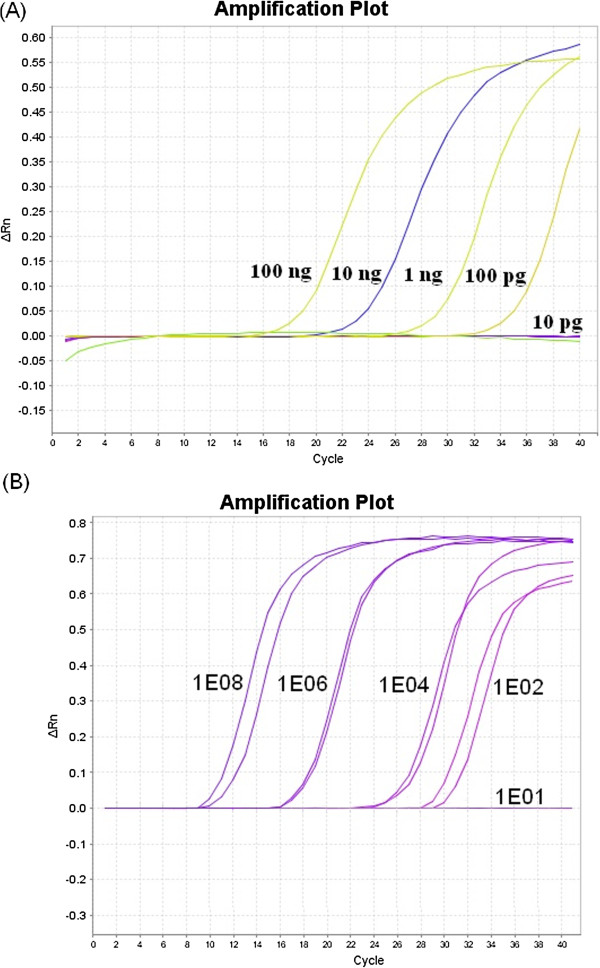
**Sensitivity determination of the developed real-time PCR assay.** (**A**) Amplification in samples containing successive 10-fold dilutions of CoHV-1 standard isolate. Concentration of DNA in each sample was given. (**B**) Amplification in samples containing serial dilutions (10^8^ – 1E8, 10^6^ – 1E6, 10^4^– 1E4, 10^2^ – 1E2 and 10^1^ – 1E1) copies of pGW1126 plasmid. ΔRn is an increment of fluorescent signal during successive cycles.

The designed primers used in real-time PCR did not allow for the amplification of DNA templates extracted from MDV, HVT, PiCoV and FadV-1 strains. Therefore, we considered them specific only for the DNA-dependant DNA polymerase gene of CoHV-1. The method enabled detection of 100 pg of standard GW1126 strain or 10^2^ copies of pGW1126 plasmid with the concentration determined as 1.32 μg/μL and was considered as very sensitive (Figure 2). Following the study, all 88 DNA templates from the propagated field strains were examined with the developed method. Eighteen samples (20.4%) were positive for CoHV-1 (Table 1). The highest cycle threshold (C_T_) values – 17.1 and 21.7 – were found in rock pigeons from the city of Lublin (GJW1130 and GJW1126). Lower values (C_T_ ranging from 31.1 to 36.1) were found in free-ranging birds like Peregrine Falcon (*Falco peregrinus*) – strain GJW1122, Herring Gull (*Larus argentatus)* – strain GJW1124 or Tawny Owl (*Strix aluco*) – strain GJW1138.

**Table 1:** GenBank accession numbers and origin of DNA sequences analysed in the study

**Accession number**	**Strain**	**Source of isolation/reference virus stock**
Genbank:JX892992	GJW1122	Peregrine Falcon (*Falco peregrinus*)
Genbank:JX892993	GJW1123	Common Buzzard (*Buteo buteo*)
Genbank:JX892994	GJW1124	Herring Gull (*Larus argentatus*)
Genbank:JX892995	GJW1125	Eurasian Goshawk (*Accipiter gentilis*)
Genbank:JX892997	GJW1126	Rock Pigeon (*Columba livia*)
Genbank:JX892998	GJW1127	Common Buzzard (*Buteo buteo*)
Genbank:JX892999	GJW1128	Grey Heron (*Ardea cinerea*)
Genbank:JX892996	GJW1129	Rock Pigeon (*Columba livia*)
Genbank:JX893000	GJW1130	Rock Pigeon (*Columba livia*)
Genbank:JX893001	GJW1131	Rock Pigeon (*Columba livia*)
Genbank:JX893002	GJW1132	Peregrine Falcon (*Falco peregrinus*)
Genbank:JX893003	GJW1133	Rock Pigeon (*Columba livia*)
Genbank:JX893004	GJW1134	Hooded Crow (*Corvus cornix*)
Genbank:JX893005	GJW1135	Eurasian Goshawk (*Accipiter gentilis*)
Genbank:JX893006	GJW1136	Rock Pigeon (*Columba livia*)
Genbank:JX893007	GJW1137	Eurasian Kestrel (*Falco tinnuncuclus*)
Genbank:JX893008	GJW1138	Tawny Owl (*Strix aluco*)
Genbank:JX893009	GJW1139	Song Thrush (*Turdus philomelos*)
Genbank:AF131890	KP 21/23	Rock Pigeon (*Columba livia*)
Genbank:GQ478232	PO1W124	Southern Boobook (*Ninox novaeseelandiae*)
Genbank:JF810822	08190111	Human herpesvirus 1
Genbank:AF168792	SA-2	Gallid herpesvirus 1
Genbank:NC002577	HPRS24	Gallid herpesvirus 3
Genbank:NC005264	97-0001	Psittacid herpesvirus 1
Genbank:HSKUL30H	GA	Gallid herpesvirus 2
Genbank:AF291866	FC126	Meleagrid herpesvirus 1

The species distribution among the 88 examined birds has shown that the most frequently CoHV-1 was detected in Peregrine Falcons (2/2), rock pigeons (6/11) and Common Buzzards (2/11). Interestingly, CoHV-1 was identified for the first time in Herring Gull, Grey Heron (*Ardea cinerea)*, Hooded Crow (*Corvus cornix*) and Song Thrush (*Turdus philomelos*). Seven out of seventeen examined species of birds were free from CoHV-1 infection (Table 2).

**Table 2:** Distribution of CoHV-1 among 88 birds received for the WNV surveillance program

**Source of isolation**	**Total number of examined birds**	**Number of CoHV-1 positive birds**
Peregrine Falcon (*Falco peregrinus*)	2	2
Common Buzzard (*Buteo buteo*)	11	2
Herring Gull (*Larus argentatus*)	4	1*
Eurasian Goshawk (*Accipiter gentilis*)	19	2
Rock Pigeon (*Columba livia*)	11	6
Grey Heron (*Ardea cinerea*)	2	1*
Hooded Crow (*Corvus cornix*)	1	1*
Eurasian Kestrel (*Falco tinnuncuclus*)	4	1
Tawny Owl (*Strix aluco*)	6	1
Song Thrush (*Turdus philomelos*)	1	1*
Bohemian Waxwing (*Bombycilla garrulous*)	2	0
Long-eared Owl (*Asio otus*)	1	0
Mallard (*Anas platyrhynchos*)	8	0
Common Pochard (*Aythya ferina*)	1	0
European white stork (*Ciconia ciconia*)	12	0
White-tailed Eagle (*Haliaeetus albicilla*)	2	0
Great Cormorant *(Phalacrocorax carbo*)	1	0

The species, number and the result of the CoHV-1 study are given. The asterix “*” indicates the bird species which the CoHV-1 infection was detected for the first time in this study.

### DNA sequencing

The obtained PCR products are 207 bp long. The accession numbers of 18 new CoHV-1 sequences were shown in Table 1.

### Phylogenetic analysis

**Figure 3:**
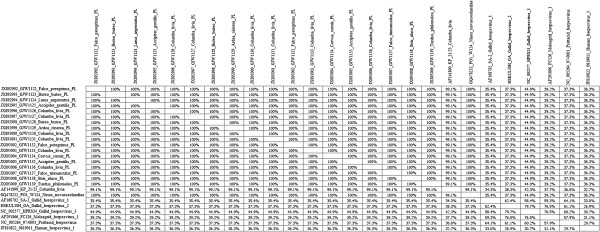
**Nucleotide sequence matrix similarity of 26 aligned sequences composed from 18 Polish field strains of CoHV-1 and 8 GenBank database accessible sequences.** The similarity of each sequence is compared and given as percentage.

**Figure 4:**
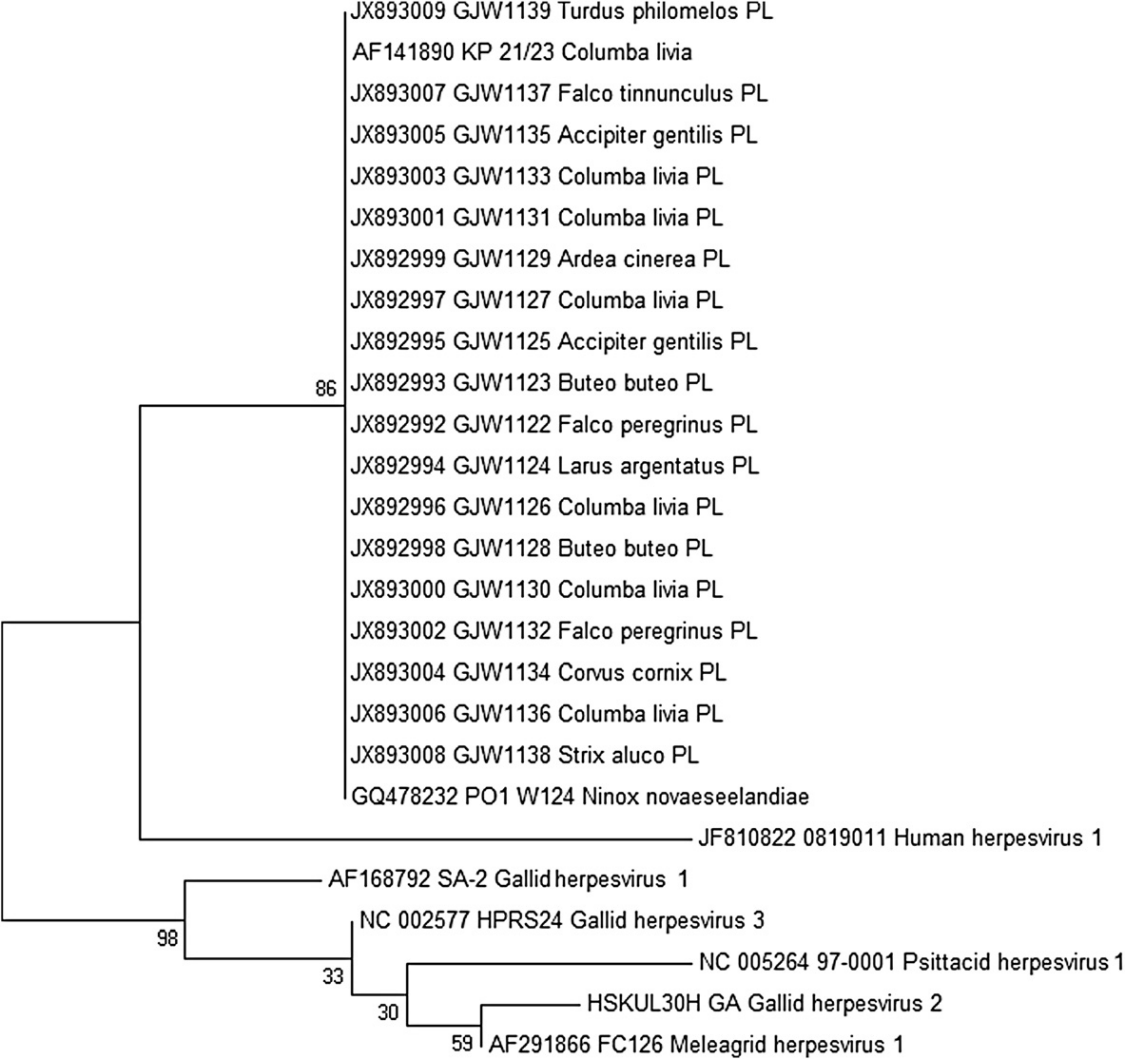
**Phylogenetic tree constructed on the basis of the conducted alignment of the DNA-dependent DNA polymerase from Polish field CoHV-1 strains and herpesviruses derived from GenBank database.** BOOTSTRAP values are indicated.

The conducted Genious™ sequence alignment for the total 26 herpesvirus sequences showed a 100% identity within the group of Polish CoHV-1 strains. This similarity was independent from host species. These strains sequence was identical with the reference sequence of PO1 W124 strain originating from Australia. Minor differences within CoHV-1 sequence were found in KP 21/23 strain isolated in Germany since its identity to other examined CoHV-1 strains reached 99.1%. CoHV-1 strains showed an identity from 37.3 to 44.9% with three other compared Alphaherpesviruses of poultry (*Gallid herpesvirus* 2, *Gallid herpesvirus 3* and *Meleagrid herpesvirus* 1). The identity of CoHV-1 strains to ILTV (*Gallid herepesvirus 1*) was lower and reached from 34.3 to 35.4%. Interestingly, PsHV-1 affecting parrots showed an identity of 37.3% with the examined CoHV-1 strains. Finally, the analysed CoHV-1 strains shared an identity from 32.7 to 36.3% with the related human herpesvirus 1 (Figure 3). On the basis of the multiple alignment, analysis and neighbour-joining (NJ) computational method, the phylogenetic tree was constructed (Figure 4). All CoHV-1 strains fall into a single group. The second group was represented by a single human herpesvirus 1 whilst the third less homogeneous group was composed of sequences of poultry herpesviruses. The BOOTSTRAP values exceeded 86 between the main created groups. Within the group of poultry herpesviruses, these values were lower and reached from 30 to 59, respectively. The conducted analysis confirmed the data obtained by similarity matrix data (Figure 3). In general, the sequences of the examined CoHV-1 strains are highly homogenous as this was found on the basis of similarity and phylogenetic analysis. The comparison with two other CoHV-1 homological sequences from GenBank has shown that herpesviruses of pigeons and free-ranging birds present a high similarity that is independent from the species or geographical origin.

## Discussion

Columbid herpesvirus (CoHV-1) has been primary described in 1940 by Smadel *et al*. [3] as an agent affecting pigeons that caused visceral lesion such as necrosis especially in liver. Herpesvirus infection was frequently identified in young pigeons and free-ranging birds from different countries of Europe [12,16,20-22], Iraq [23], USA [1,13,15], Australia [4,14] and Canada [5]. The virus is antigenically related to CoHV-1 and was found in falcons and owls [1,4,5,12-16].

The current study has shown that eighteen samples (20.4%) were positive for the CoHV-1 presence. The applied real-time PCR method was highly sensitive which facilitated the collection of reliable results on the CoHV-1 proportion in Poland. The most frequent CoHV-1 was found in *Falconidae*, *Strigidae* and *Accipitridae*. In other reports from the United Kingdom conducted by Zesivanovitis *et al*. [24] the prevalence of CoHV-1 ranged from 4.9% within the *Strigidae* to 8.8% in the *Accipititridae* family. The same study showed CoHV-1 seroprevalence among free-ranging owls reaching 8.2%. The analytic method selection might explain this variation between the percentages of CoHV-1 positive birds since PCR-based techniques enable a more sensitive detection of viral pathogens in comparison to serological assays.

The CoHV-1 affected birds may show other non-specific signs like depression, diarrhoea and neurological signs, but this may be also related with other co-infections with adeno- (FadV), circo- (PiCV) and paramyxovirus aPMV-1 [17,25]. However, in the past two variants of neuropathogenic CoHV-1 strains occurred in rock pigeons from Iraq and Poland [22,26]. The specific lesions include hepatic inclusion bodies with focal necrosis in spleen and pancreas [1,4,5,12-14,16]. Meanwhile, there are many asymptomatic infections in pigeons older than 6 months. The reservoirs of CoHV-1 are latently infected pigeons and the environment, in which the virus may remain infectious for a long time. The latter is more convincing when taking into account the survival of other Alphaherpesviruses such as MDV which may remain infectious at least for 8 months in 22-25°C and for 3 years in 4°C [18]. Free-ranging pigeons are thought to transmit the virus to domestic pigeons [2]. In addition, the meat may be infectious for birds of prey that hunt these. We presume that Herring Gulls as scavengers may be infected by consumption of dead pigeons, or when they share food with pigeons in the cities. In our study, we have shown that also free-ranging non-raptorial birds may be infected with CoHV-1. We have also observed neurological signs in Peregrine Falcon female. The absence of WNV, AIV and aPMV-1 in addition to the presence of CoHV1 in the brain of this Peregrine Falcon with central nervous signs indicates a possible neuroinvasive form of herpesvirus infection, such as those described previously.

What is worth mentioning, the samples of brain are not commonly used for CoHV-1 isolation and detection. However, our study has shown that the proportion of CoHV-1 infection was even higher in comparison to previous reports [26]. The comparison of different detection methods is always very difficult because serological assays investigate the immunological response of the host to the pathogen whereas our real-time PCR method revealed the presence of DNA what does not determine the detection of fully functional virus and possible viremia. The complete investigation should include full necropsy and histological assay which we were unable to conduct. However, virus isolation in cell cultures confirmed the CoHV-1 presence. So far the methods of CoHV-1 detection included mainly histopathological examinations which is dedicated only to experienced pathologists, serum neutralisation tests, DNA endonuclease digestion and polymerase chain reaction (PCR) which in spite of their usefulness are laborious and time-consuming [7,9,10,13]. The uniplex-PCR technique facilitating the detection of all three most important viral pathogens of pigeon was further described by Frieck *et al.*[25]. The problem with CoHV-1 detection may also be caused by latent infection that is specific for *Herpesviridae* infecting pigeons and owls [1,4,5]. The present study describes the use of SYBR Green I – based real-time PCR technique, which provides fast and reliable results on the CoHV-1 infection of both pigeons and free-ranging birds. The design reason of this technique was to accelerate, simplify and increase the sensitivity of CoHV-1 detection. The main advantage of our technique is also the lower risk of false negative results as it is important due to the kind of used samples in this study. On the other hand, the main drawback of the used method was CoHV-1 detection after the culturing of CoHV-1 strains, thus our results lack quantification of the virus in the original brain samples. It would also be valuable to examine samples from other organs including liver and spleen in the future. It is well known that real-time PCR may not provide any information about the cause of the observed clinical signs. However, it is valuable in case of latent infections which cannot be identified by histological assays. The best combination would be the use of a histopathological assay together with our real-time PCR that may facilitate sensitive herpesvirus detection and provide valuable remarks on the potential death cause.

We presume that the obtained proportion of CoHV-1 in free-ranging birds may be slightly underestimated due to the kind of used samples. The results obtained in our study confirm previous findings that all recently reported herpesviruses from birds of prey belong to CoHV-1 [1,5,13,14,27]. Indeed, herpesviruses are not restricted to a specific host or tissue and may cross the barriers of the specific host. Our study has shown that the sequence identity of 18 CoHV-1 Polish strains was 100% independently from the species of the affected bird. The analysed 207 bp fragment was conserved among all examined Polish strains of CoHV-1. Consistently, CoHV-1 sequences identical to other examined herpesvirus strains have been found for the first time in four species of non-raptorial birds including: Herring Gull (*Larus argentatus*), Grey Heron (*Ardea cinerea*), Hooded Crow (*Corvus cornix*) and Song Thrush (*Turdus philomelos*). A similar identity (99.1-100%) was found by comparing examined CoHV-1 to two other foreign sequences originating from Germany and Australia. The identity of CoHV-1 sequences seems to be independent from species and geographical origin. This may be also caused by the low number of relevant CoHV-1 sequences accessible in the GenBank database. Interestingly, CoHV-1 strains shared at least 37.3% similarity with other related herpesviruses isolated from chickens and turkeys. In addition, the PsHV-1 that infects parrots has the same identity level, which may confirm the homology of the particular genes of Alphaherpesviruses. The related human herpesvirus 1 falls into a distinct group, but still its identity to CoHV-1 strain reached from 32.7 to 36.3% that implies on at least partially conservative character of the DNA-dependant DNA polymerase. The presented phylogenetic data were reliable in spite of three lower BOOTSTAP values estimated for *Gallid herpesvirus 3*, *Psittacid herpesvirus 1* and *Gallid herpesvirus 2* strains.

## Conclusion

In concluding the conducted study, we have shown that the sequence of DNA-dependent DNA polymerase of 18 Polish isolates has shown 99.1% identity independently from their species and geographical origin. This may suggest a possible transmission of the virus between population of domestic and free-ranging birds in Poland. Interestingly the same isolates were found in domestic pigeons, raptors and non-raptorial birds as may be the evidence on close similarity between herpesviruses. Further studies including cloning of the whole CoHV-1 genome and its detailed analysis will hopefully explain the role of the observed similarities and differences in the genome.

## Abbreviations

NVRI: National veterinary research institute, Pulawy, Poland; CoHV-1: Columbid herpesvirus 1; PsHV-1: Psittacid herpesvirus 1; MDV-1: Marek’s disease virus serotype 1 (*Gallid herpesvirus 2*); MDV-2: Marek’s disease virus serotype 2 (*Gallid herpesvirus 3*); ITLV: Infectious laryngotracheitis virus 1 (*Gallid herpesvirus 1*); HVT: Herpesvirus of turkey (*Meleagrid herpesvirus 1*); WNV: West Nile Virus; AIV: Avian influenza virus; CEF: Chicken embryo fibroblasts; SPF: Specific pathogen free; MEM: Minimal essential medium; w/v: Weight by volume percentage; PCR: Polymerase chain reaction; h p. i.: Hours post-infection; μL: Microliter; pg: Pico gram (10^-12^ g); CT: Cycle threshold value; SYBR Green I: Real-time PCR assay kit containing SYBR Green I dye binding to double-strand structures of DNA; BOOTSTRAP: Statistical method in phylogenetics used to estimate an error during computation of relation between similar nucleotide sequences at phylogenetic tree

## Competing interests

The authors declare that they have no competing interests.

## Authors’ contributions

Most of the experiments were conducted by WJG who conducted viral materials preparation, DNA extraction, real-time PCR development and sequence analysis. ESS assisted in experimental design of the study. SP provided infectious materials from falcons with detailed description and documentation. PW and ST provided materials for the study from herpesvirus infected pigeons. HM provided additional infectious materials from non-raptorial and raptor birds. ESS, SP and PW and HM participated in the coordination of the study. WJG wrote this manuscript and prepared figures for publication. The final manuscript was read and approved by all the authors.
